# A novel mutation in *SoIAA20* confers cross‐resistance to 2,4‐Dichlorophenoxyacetic acid and other auxinic herbicides in *Sonchus oleraceus*

**DOI:** 10.1002/ps.8413

**Published:** 2024-09-13

**Authors:** Mahima Krishnan, Tijana Petrovic, Julian G. Schwerdt, Alicia B. Merriam, James P. Hereward, Christopher Preston

**Affiliations:** ^1^ School of Agriculture, Food and Wine University of Adelaide Glen Osmond Australia; ^2^ School of Biological Sciences University of Queensland Brisbane Australia

**Keywords:** 2,4‐D resistance, Aux/IAA, degron mutation, reduced herbicide translocation

## Abstract

**BACKGROUND:**

2,4‐Dichlorophenoxyacetic acid (2,4‐D) and other auxinic herbicides are important for weed control in cropping systems globally. Weeds with resistance to 2,4‐D and other auxinic herbicides have evolved, including several populations of *Sonchus oleraceus* from multiple sites in Australia. We report the underlying mechanism in these populations that gives rise to auxinic herbicide resistance.

**RESULTS:**

We studied a total of three susceptible and eight resistant *Sonchus oleraceus* populations. All resistant populations had a deletion of three amino acids flanking the degron sequence of an *Aux/IAA* gene, *SoIAA20*, which was not found in the three susceptible populations. The eight populations with the resistant allele were also resistant to dicamba, fluroxypyr and clopyralid. The resistant plants also had reduced movement of 2,4‐D out of the treated tissues compared to susceptible plants.

**CONCLUSION:**

The paired deletion flanking the degron region of *SoIAA20* likely provides resistance to 2,4‐D by restricting the movement of 2,4‐D from the treated tissue to the rest of the plant. We hypothesise that this deletion keeps the 2,4‐D bound to the target site. © 2024 The Author(s). *Pest Management Science* published by John Wiley & Sons Ltd on behalf of Society of Chemical Industry.

## Graphical abstract

Several populations of *Sonchus oleraceus* were found to be highly resistant to 2,4‐Dichlorophenoxyacetic acid (2,4‐D), with cross‐resistance to other auxinic herbicides. The resistant populations all contained three amino acid deletions on either side of the highly conserved degron of *SoIAA20*, whereas the susceptible populations did not. We hypothesise that these deletions affect the binding dynamics of the 2,4‐D, SCF^TIR1/AFB^ and SoIAA20, and trap the 2,4‐D, preventing its release to move throughout the plant.

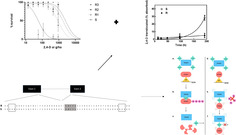

## 1 INTRODUCTION

*Sonchus oleraceus* (sowthistle or common sowthistle) is a major weed of many fallow and cropping systems, including vegetables and grains.^1^, ^2^ It is a prolific producer of highly mobile seed, which are wind‐dispersed. *S. oleraceus* seeds have low dormancy and germinate on the soil surface.^1^, ^3^ Most grain cropping systems in Australia are no‐till, which provides a favourable environment for the establishment of seedlings.^1^, ^3^, ^4^ *S. oleraceus* interferes with crop productivity by competing for resources, using soil moisture during fallow periods, and acting as a reservoir of pests and diseases.^5^ Early management strategies of *S. oleraceus* in grain production systems involved reliance on group 2 herbicides, such as chlorsulfuron, to which this species readily evolved resistance.^6^ This has placed mounting pressure on the efficacy of group 4 herbicides, such as 2,4‐Dichlorophenoxyacetic acid (2,4‐D), which have been largely successful in the control of common *S. oleraceus*. However, the evolution of resistance to this mode of action was inevitable.^6^

There was a widespread assumption that resistance to auxin mimics, like 2,4‐D, would be slow to evolve. The efficacy of 2,4‐D has stood in contrast to the accelerating resistance to glyphosate with current statistics listing 25 species resistant to 2,4‐D and 58 species resistant to glyphosate.^7^, ^8^, ^9^ The adoption of glyphosate tolerant crops with the consequent widespread use of glyphosate has greatly increased the selection pressure for glyphosate resistant weeds.^10^ To address this, 2,4‐D and dicamba tolerant crops have been commercialised. There is evident risk that the rising adoption of 2,4‐D tolerant crops will place further selection pressure for resistance to this herbicide.^10^

Despite the widespread use of 2,4‐D, the mechanism of action is still not fully understood. Recent work points to mutations in different members of the *Aux/IAA* gene family causing resistance to dicamba and 2,4‐D, respectively, which suggest that these critical players in the auxin perception and response pathway are also important in the action of auxinic herbicides.^11^, ^12^, ^13^ Under low auxin levels, Auxin/indole‐3‐acetic acid (Aux/IAA) are bound to transcription factors called auxin response factors (ARF), which are, in turn, bound to genetic elements called auxin response elements (AuxRE). When auxin levels increase above a threshold in the cell, a signal cascade is triggered whereby the SCF^TIR1/AFB^ complex, by binding auxin, recruits the Aux/IAA for proteasomal degradation, thereby releasing the ARF to activate the AuxRE.^14^, ^15^ This interaction is mediated, among others, through a highly conserved motif found in majority of Aux/IAA proteins called the degron that has a highly conserved GWPPV core.^16^, ^17^ Le Clere *et al*. and Figueiredo *et al*. identified mutations in or proximal to the degron region that have resulted in the destabilising of interactions between the relevant Aux/IAA and their respective SCF^TIR1/AFB^ complexes. LeClere *et al*. and Figueiredo *et al*. propose that this has likely inhibited the ubiquitylation of the Aux/IAA by the SCF^TIR1/AFB^, a signal tag that usually destines any protein that bears it for proteasomal degradation. This reduces or prevents the usual degradation rate of the relevant Aux/IAA. However the mutation might work to confer auxin herbicide resistance, it has been confirmed that this mutation is sufficient to confer 2,4‐D and dicamba resistance in plants that have been transformed with the resistant *Aux/IAA* allele.

The present study aims to identify the 2,4‐D resistance mechanism in *S. oleraceus*. We studied a total of 11 populations of *S. oleraceus* collected from different sites in South Australia, Victoria, and New South Wales, Australia, including three susceptible and eight resistant populations. The resistance profiles of the populations to 2,4‐D and related auxinic herbicides, along with herbicide translocation, metabolic detoxification and the sequencing of the *Aux/IAA* gene family were investigated.

## 2 MATERIALS AND METHODS

### 2.1 Plant material

Three putative 2,4‐D resistant populations were collected from clover seed production fields in the south‐east of South Australia in 2014, where 2,4‐Dichlorophenoxy)butyric acid (2,4‐dB) had been repeatedly used. These populations were sent in from three different farmers to test and confirm 2,4‐D resistance. A susceptible population was collected from Garden Island, South Australia, a recreational area where herbicides are not used. Following characterisation of 2,4‐D resistance profiles of the initial susceptible and three resistant populations, further *S. oleraceus* populations were recruited by screening with 228 g ha^−1^ of 2,4‐D that would distinguish susceptible from resistant populations. These populations were then used for genotyping. Three additional populations were from northern New South Wales, previously described in Cook *et al*.,^18^ and comprised two susceptible and one resistant population based on survival at 228 g ha^−1^ of 2,4‐D and genotyping. Another four populations were from a 2017 *S. oleraceus* survey in the south‐east region of South Australia and were all confirmed resistant based on survival at 228 g ha^−1^ of 2,4‐D and genotyping. Further details of the populations used in this study are provided in Table S1.

### 2.2 Dose response

Seeds were germinated on cocopeat potting mix, transplanted into pots (8.5 × 9.5 × 9.5 cm) and grown in shadehouses. There were nine pots per population, each with four plants. A cabinet sprayer with a mobile boom containing Teejet flat fan nozzles (TeeJet 110 015; TeeJet Technologies, Victoria, Australia) calibrated to 118 L ha^−1^, output at 300 kPa was used for herbicide application. Plants were sprayed at the four to five leaf stage with rates of 14, 28, 57, 114, 228, 455 and 910 g ha^−1^ for the first experiment for 2,4‐D; 7, 14, 28 and 57 g ha^−1^ for the S population, and 114, 228, 455 and 910 g ha^−1^ for the three R populations for the second experiment for 2,4‐D; 15, 30, 60, 120, 240 and 480 g ha^−1^ for chlopyralid; 35, 70, 140, 280 and 560 g ha^−1^ for dicamba; 12.5, 25, 50, 100 and 200 g ha^−1^ for fluoroxypyr; and 12, 24, 48, 96 and 192 g ha^−1^ for triclopyr. All dose response experiments were run twice. Survival was assessed 6 weeks after treatment. Both susceptible and resistant plants displayed strong epinasty. Survivors were counted as those that had new meristematic growth. Those that did not have meristematic growth eventually died. Data were analysed using PriProbit (version 1.63) to calculate LD_50_ values (Table 1).^19^ Repeat experiments were analysed by two‐way ANOVA and, when the experimental run was not significant, data were pooled across experiments prior to probit analysis.

**Table 1:** 2,4‐dichlorophenoxyacetic acid LD_50_ (g ha^−1^) values with 95% confidence intervals in parentheses and resistance index of S and R populations

Population	Experiment 1 (*n* = 4)	Resistance index (R/S ratio) Exp 1	Experiment 2 (*n* = 9)	Resistance index (R/S ratio) Exp 2
S	58.55 (30, 102)		76.96 (52, 145)	
R1	1787.5 (734, 7937)	30	1860.5 (976, 5245)	24
R2	2592.9 (843, 17 155)	44	1450.8 (945, 2905)	19
R3	894.59 (459, 2434)	15	549.25 (407, 806)	7

*n*, number of plants per treatment.

To identify potential cytochrome P450 (CYP450)‐mediated detoxification of 2,4‐D, plants were treated with 1000 g ha^−1^ a.i. of malathion 24 h prior to treatment with 14, 28, 57, 114, 228, 455, 910, 1365, 1820 and 2275 g ha^−1^of 2,4‐D.^20^ A sum‐of‐squares F‐test was done for the curves of the populations with and without malathion to ascertain any significant effects of malathion in GraphPad Prism 10.0.0 (www.graphpad.com). Malathion is a well‐established inhibitor of some CYP450 enzymes in plants.^21^

### 2.3 Translocation

Absorption and translocation studies of 2,4‐D in the susceptible and resistant populations were conducted following the methods described in Dang *et al*.^22^ Twelve plants of each population were sampled at 24, 48 and 72 h after treatment (HAT). The percentage of radioactivity was calculated as the amount present in the wash solution, plus plant parts as a percentage of that applied. The amount of 2,4‐D absorbed was calculated as the amount of radioactivity present in plant parts as a percentage of the radioactivity recovered. Translocation of 2,4‐D was calculated as the amount of radioactivity present in individual plant parts as a percentage of that absorbed. A two‐way ANOVA was performed on the initial translocation experiment to study whether there were any differences between the different populations at 72 HAT (Table S2). A second experiment was then carried out with just the S and R2 populations. Three plants each of S and R2 were harvested at 24, 48, 96 and 192 HAT. Calculations were performed as described above.

### 2.4 Whole‐genome sequencing and assembly of *S. oleraceus*

We used the *S. oleraceus* genome available from the international weed genomics consortium data portal to identify *AUX/IAA* genes (https://weedpedia.weedgenomics.org/jbrowse/50207/635). This genome was obtained from a glyphosate resistant line of *S. oleraceus* derived from a sample collected in the Liverpool Plains area of New South Wales (Australia) soon after glyphosate resistance was first detected in 2008.^18^

### 2.5 Prediction of putative *Aux/IAA* genes

Targeted gene annotation was performed using AUGUSTUS.^23^ AUGUSTUS was used because at the time of analysis no annotation was available. Full alignments for the PF02309 (AUX/IAA) family were downloaded from the InterPro protein family database (https://www.ebi.ac.uk/interpro/ ^24^) and converted to an AUGUSTUS profile using internal tools. AUGUSTUS‐PPX was used to search the assembled *S. oleraceus* genomic contigs and candidate coding regions assessed by homology to well‐characterised Aux/IAA sequences from Ensembl Plants (https://plants.ensembl.org ^25^).

### 2.6 Sequencing of putative *Aux/IAA* genes

Fresh leaf material was collected and snap frozen in liquid nitrogen. RNA was extracted using a Qiagen RNA extraction kit, in accordance with the manufacturer's instructions. DNA contamination was removed by treating the RNA with DNase (Ambion, ThermoFisher) and cDNA was synthesised using oligoDT primers and the Tetro cDNA synthesis kit (Millenium Bioscience Pty Ltd.) in accordance with the manufacturer's instructions. Polymerase chain reactions were conducted on ~200 ng of cDNA with MyFi DNA polymerase (Millenium Bioscience Pty Ltd.), in accordance with the manufacturer's instructions, with 55 °C for annealing and 1 min and 30 s for the extension time. Primers for all *Aux/IAA* candidates were designed to have annealing temperatures of 55 °C and bind close to the start and end of the predicted coding sequence (Table S3). PCR products were then sequenced by Sanger sequencing method at the Australian Genome Research Facility. Sequences of the different *Aux/IAA* were aligned with their respective predictions and studied for any differences in sequences between putative R and S populations. A BLAST was performed for each gene member of which *Lactuca sativa Aux/IAA* genes had the highest bit‐score match (Table S3). The *SoIAA20* was named based on the closest *Lactuca sativa* match in a UPGMA tree generated in Geneious Prime 2023 (http://www.geneious.com/) (Fig. 3).

## 3 RESULTS

### 3.1 Survival to 2,4‐D

**Figure 1:**
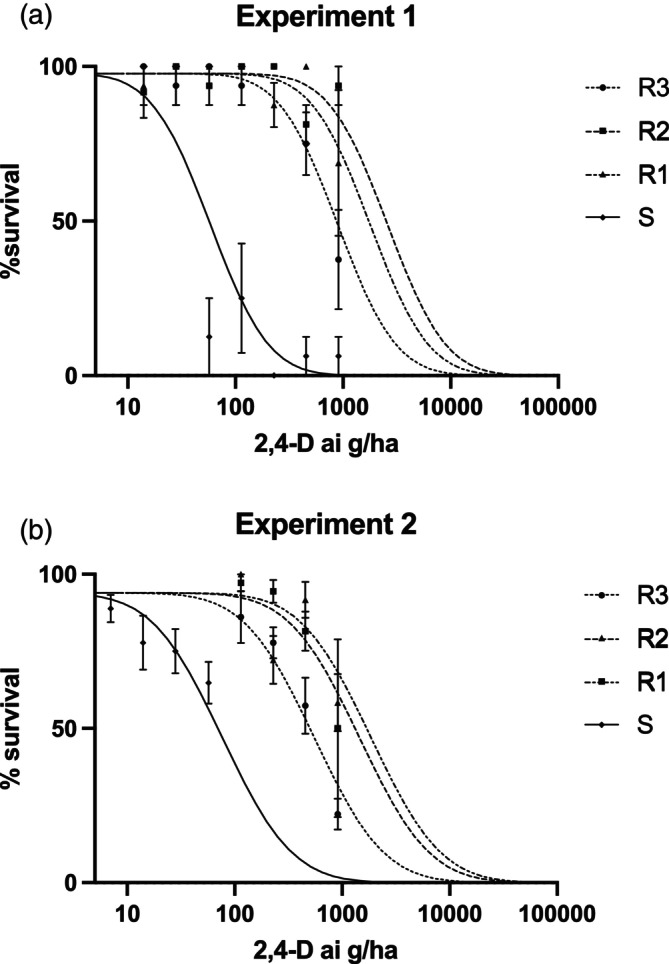
Dose response curves for the survival of the S and three R populations. The dose response experiment was repeated: (a) experiment 1, with each data point being the mean of four replicates, and (b) experiment 2, with each data point being the mean of nine replicates. Error bars are standard error of the mean (SEM). Lines are the predicted responses from the Probit analysis. 2,4‐D, 2,4‐dichlorophenoxyacetic acid.

The susceptible population was controlled at 100 g ha^−1^ of 2,4‐D (Table 1) in contrast to the resistant populations, which could not be fully controlled at the highest administered dose of 910 g ha^−1^. The resistant populations were between 10‐ and 30‐fold more resistant to 2,4‐D compared to the susceptible population. The R2 population was at the highest end of the resistance spectrum (Fig. 1(a)) with an LD_50_ value of 2600 g ha^−1^ in the first experiment but was the second most resistant population in the second 2,4‐D trial (Fig. 1(b)), still having a high LD_50_ value of 1400 g ha^−1^. Population R1 had the second highest resistance in the first trial (Fig. 1(a)) with an LD_50_ of 1800 g ha^−1^ and had the highest resistance in the second trial (Fig. 1(b)) with an LD_50_ of 1900 g ha^−1^. Population R3 had consistently lower LD_50_ compared to R1 and R2 and was 7‐ to 15‐fold resistant compared to the susceptible population. The recommended label rate of 2,4‐D for thistles (including *S. oleraceus*) is 350 g ha^−1^ (Nufarm label for 2,4‐D Amine 625). The LD_50_ values (Table 1) for R1, R2 and R3 exceed this rate.

### 3.2 Survival to other auxinic herbicides

The three putative resistant populations were also resistant to the other group 4 herbicides clopyralid, dicamba and fluroxypyr when compared with the susceptible population, although the levels of resistance were not as high compared with 2,4‐D (Table 2). Resistant populations R1 and R2 were consistently more resistant to clopyralid and fluroxypyr than population R3, which had lowest resistance. Resistant population R2 was most resistant to dicamba with R1 and R3 having lower levels of resistance (Table 2).

**Table 2:** LD_50_ values of S and R populations with 95% confidence intervals in parentheses, in experiments with clopyralid (*n* = 7), dicamba (*n* = 7) and fluroxypyr (exp1 *n* = 4; exp2 *n* = 3)

Herbicide	Experiment	S	R1	R2	R3
		LD_50_ with 95% confidence intervals^b^			
Clopyralid	Pooled	52 (42, 64)	243 (187, 360)	205 (161, 263)	89 (68, 109)
Dicamba	Pooled	100 (85, 118)	242 (194, 306)	490 (299, 745)	246 (90, 748)
Fluroxypyr	1	59 (47, 77)	164 (110, 404)	89 (69, 117)	79 (63, 100)
	2	51 (39, 65)	>200^b^	>200^b^	243 (158, 868)

Pooled data are from two experiments. *n*, number of plants per treatment.

### 3.3 Potential metabolic resistance

Resistance through metabolic detoxification of the herbicide can often be reversed with malathion, a known P450 inhibitor, prior to treatment with the herbicide.^20^ The survival rates of the resistant populations were not reduced when treated with malathion prior to 2,4‐D application, although there were differences in the S population, indicating that the resistance may not be due to P450‐based metabolic detoxification, although other detoxification enzymes could be implicated (Table S4).^26^

### 3.4 Herbicide translocation

Herbicide absorption was rapid in both populations, with 60% of the herbicide absorbed within 24 h of application. There were no significant differences in absorption of 2,4‐D between the resistant and susceptible populations.

**Figure 2:**
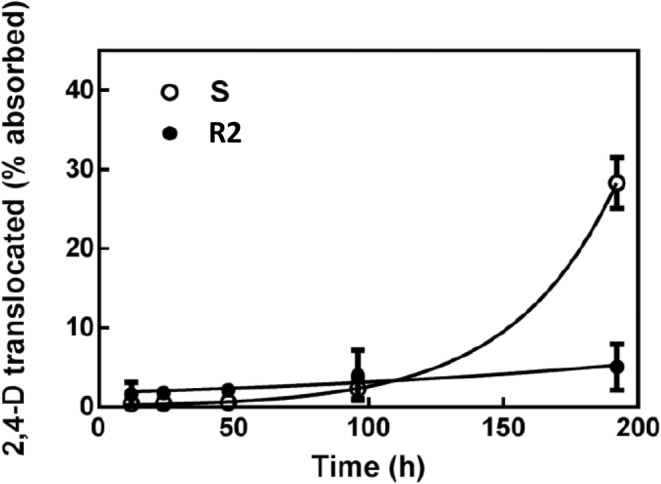
Translocation of 2,4‐dichlorophenoxyacetic acid (2,4‐D) out of the treated leaf to the rest of the plant in the S (open circle) and R2 (closed circle) populations. At each timepoint, three replicates of each population were analysed.

Translocation of the herbicide from the treated leaf to the rest of the plant was low up to 96 HAT. However, at 192 HAT 30% of the 2,4‐D had been translocated out of the treated leaf in the susceptible plants, but only 5% was translocated in the resistant plants (Fig. 2).

### 3.5 Identification of a target site mutation in a member of the *Aux/IAA* family

**Figure 3:**
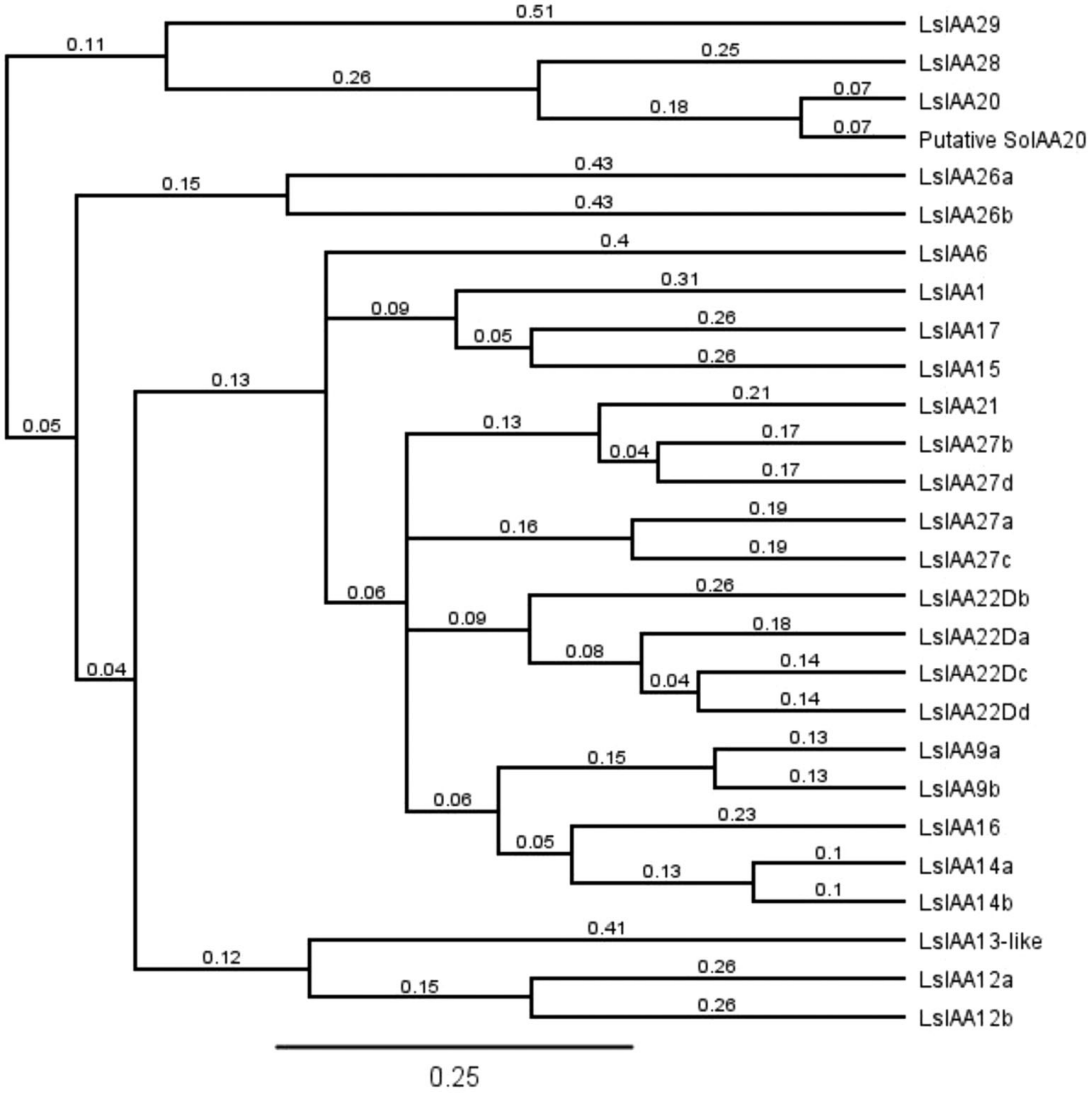
A UPGMA tree of the putative *SoIAA20* with other *Aux*/*IAA* gene members in *Lactuca sativa*. Branch labels denote amino acid substitutions per site.

**Figure 4:**
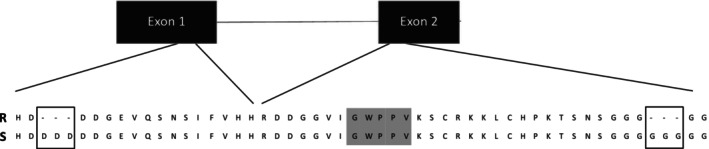
An alignment of the amino acid sequence of the R and S populations with the degron core highlighted in grey. The first deletion prior to the degron core is a result of a deletion in the first exon and the deletion following the degron is in the second exon.

There were 72 putative *Aux/IAA* genes predicted in the genome of *S. oleraceus*, with 45 of them containing the core degron motif. There was one *Aux/IAA* gene, herein designated *SoIAA20* (Genbank: PP627399.1), based on phylogeny (Fig. 3), which had two in‐frame deletions on either side of the core degron motif (Figs 4 and S1). This mutation was not present in the susceptible populations. Both deletions occur in regions containing sequence repeats. The first sequence repeat, which is deleted in the R populations, occurs in the first exon in a six aspartic acid repeat sequence, 19 amino acid residues preceding the degron core (GWPPV) region, resulting in three fewer aspartic acid residues (Fig. 4). The second deletion in the second exon occurs in a 10 glycine repeat region, 15 amino acid residues following the degron core region, resulting in three fewer glycine residues (Fig. 4). As these deletions are in‐frame, the protein is not prematurely truncated and is still theoretically functional. These two deletions were found consistently in all eight of the R populations and not in the three S populations.

## 4 DISCUSSION

### 4.1 Deletions flanking the degron are the likely basis for 2,4‐D resistance in *S. oleraceus*

Resistant populations, in contrast to the susceptible ones, contained two deletions that were three amino acids in length and flanked the degron core region of a putative Aux/IAA protein, SoIAA20 (Fig. 4). These deletions were consistently found in all eight resistant populations and not in the three susceptible ones, indicating that deletions are most likely responsible for 2,4‐D resistance and cross‐resistance to dicamba, clopyralid and fluroxypyr in this study. Previous studies in *Arabidopsis thaliana*, *Kochia scoparia* and *Sisymbrium orientale* have confirmed that mutations in or near the degron of *Aux/IAA* genes confer resistance to auxinic herbicides.^11^, ^12^, ^27^, ^28^ Resistance in *S. oleraceus* appears to be an additional example of this phenomenon. The deletions occurred in two repeat regions spread over two exons and possibly resulted from polymerase copying error.^29^ It remains unclear at this stage which of the two deletions or whether both are important for conferring 2,4‐D resistance, although both the N‐terminal and C‐terminal regions proximal to the degron are known to govern the binding affinities of the SCF^TIR1/AFB^–auxin–Aux/IAA complex.^30^

The variation in the resistance profiles of the populations in this study might indicate that different historical exposure to auxin herbicides could have affected the expression levels of the resistance phenotype, which could be tested in the future. The tetraploid nature of *S. oleraceus* also adds a layer of complexity, as there may be a dilution effect if the resistant allele was only present in one of the genomes and not in both for some of the populations.^31^ The possibility of there being another mechanism involved, perhaps downstream, that could enhance the effects of the *Aux/IAA* mutations also needs to be investigated. There could also be a mechanism that does not include the Aux/IAA family at all but could work in concert with the *Aux/IAA* mutations to confer 2,4‐D resistance. A gene inheritance study could pick apart whether this resistance is caused by a single or multiple traits.

### 4.2 Degron changes in *Aux/IAA* might mediate 2,4‐D resistance by reducing herbicide translocation

**Figure 5:**
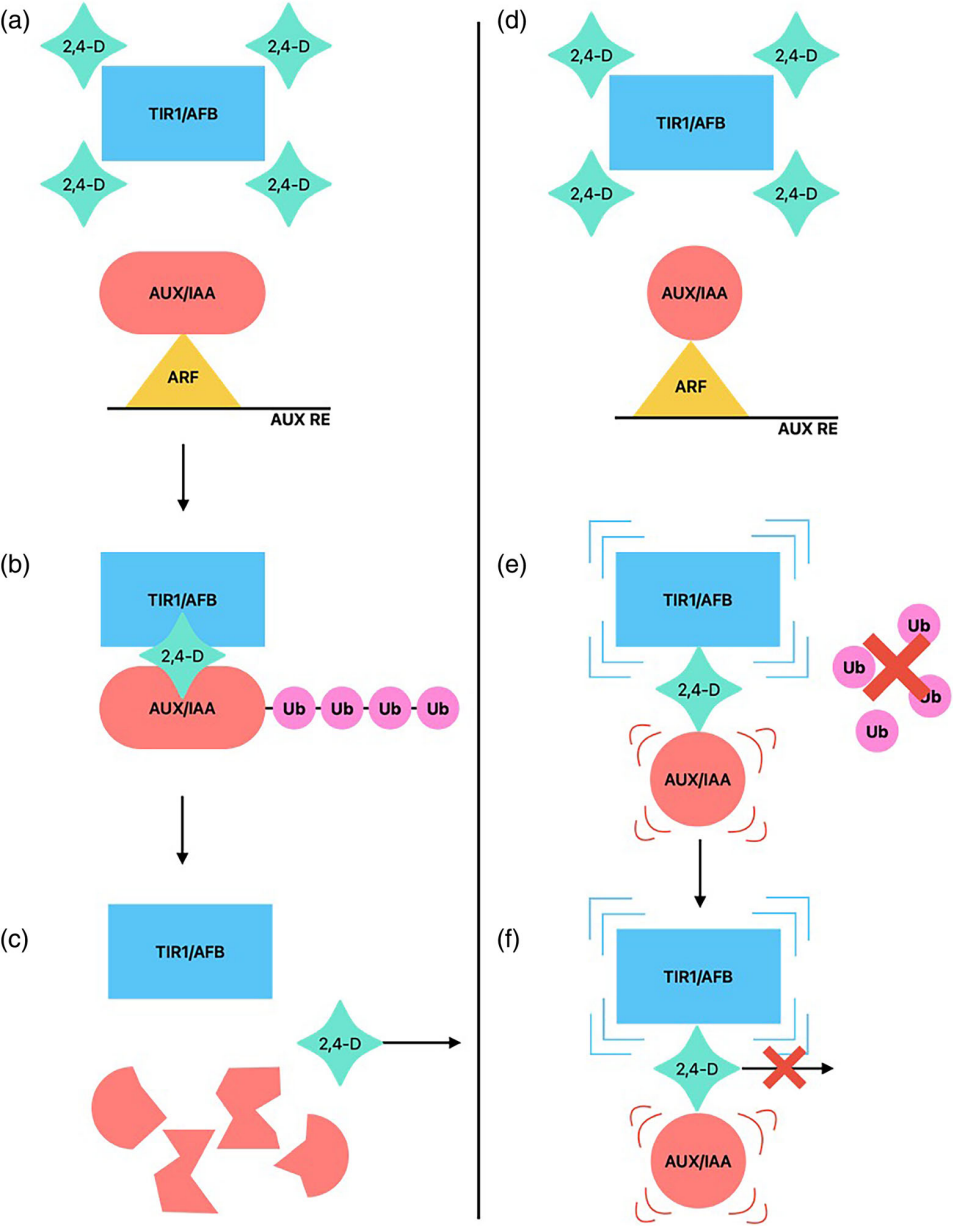
Schematic of the possible role of a mutant Aux/IAA in auxinic herbicide resistance. When the 2,4‐dichlorophenoxyacetic acid (2,4‐D) reaches a threshold in the cell it binds TIR1/AFB (a) which, in turn, recruits Aux/IAA via the degron (b) to be ubiquitylated and the eventual degradation enables the dissolution of the complex and the release of the 2,4‐D to move to other cells (c). When there is a mutation in or near the degron the TIR1/AFB along with the 2,4‐D still complexes with the Aux/IAA (d) as the degron is still present but, due to the changes in the degron region, the complex is unstable and the ubiquitylation of the Aux/IAA is hindered (e) which, in turn, prevents the degradation of the Aux/IAA that would have triggered the dissolution of the complex releasing the 2,4‐D to move to other cells (f).

We found reduced 2,4‐D translocation in the resistant plants compared to susceptible ones (Fig. 2). Reduced translocation of 2,4‐D and other auxinic herbicides has been reported in several resistant populations previously.^22^, ^32^, ^33^, ^34^, ^35^ Significantly, reduced translocation of 2,4‐D occurs in resistant populations of both *S. orientale* and *K. scoparia*, where mutations within or close to the degron region of *Aux/IAA* genes have been identified.^11^, ^12^, ^22^, ^34^ In both species, resistance was the result of a single gene trait, suggesting that reduced translocation of the herbicide was likely a consequence of the mutations in the *Aux/IAA* genes.^22^, ^36^ The degron and its flanking regions critically control the interaction between the auxins, SCF^TIR1/AFB^ and the Aux/IAA and any mutation within or near it could inhibit the proteasomal degradation of Aux/IAA, potentiating 2,4‐D resistance.^15^, ^30^ In resistant populations of *K. scoparia* and *S. orientale* the changes in and around the degron core region, respectively, destabilise the interaction between the auxinic herbicide, Aux/IAA protein and the SCF^TIR‐1/AFB^ complex.^11^, ^12^ This likely results in reduced conjugation of ubiquitin proteins on the Aux/IAA which would target the protein for proteasomal degradation, increasing the persistence of Aux/IAA. Figuereido *et al*. suggested that the reduced proteasomal turnover of Aux/IAA may result in persistent repression of ARF and the respective auxin responsive genes by Aux/IAA, causing 2,4‐D resistance. As there is reduced translocation of 2,4‐D and other auxinic herbicides in several species with mutations near the degron, including *S. oleraceus*, we suggest an alternative hypothesis, that the complex, although unstable, remains undissolved, likely leaving the herbicide trapped in the Aux/IAA‐SCF^TIR‐1/AFB^ complex^15^ (Fig. 5). The destabilised binding, while preventing the Aux/IAA from being adequately ubiquitylated, continually holds the Aux/IAA‐2,4‐D‐SCF^TIR‐1/AFB^ interaction, as the dissociation of the complex and subsequent release of the 2,4‐D is likely triggered by the degradation of Aux/IAA.^15^ This could then effectively function to trap the 2,4‐D, preventing it from translocating through the plant.^15^

Goggin *et al*. were able to simulate the reduced 2,4‐D translocation in their study's resistant populations of *Raphanus raphanistrum* by inhibiting ABCB (ATP‐binding cassette) and PIN2, which are auxin efflux transporters, in their susceptible plants. This further supports our hypothesis that 2,4‐D, persistently held by the non‐dissociating Aux/IAA‐2,4‐D‐SCF^TIR‐1/AFB^, would not be available to the efflux transporters to enable its cell‐to‐cell movement and could explain the reduced translocation seen in the resistant plants.

### 4.3 Degron mutation could cause SoIAA20 to function as a decoy for 2,4‐D

When there are multiple potential targets for 2,4‐D, the mechanism by which a mutated SoIAA20 would be adequate to act as a decoy for 2,4‐D remains unresolved. It is possible that the mutation increases affinity for 2,4‐D thereby restricting the interactions of this auxin with other Aux/IAA members. Certainly, different combinations of the TIR1/AFB and the different auxins with any of the Aux/IAA proteins exhibit different binding dynamics.^11^, ^30^ The Aux/IAA is a key determinant of the dynamics in these interactions.^37^

### 4.4 Multiple gene members provide multiple opportunities for mutations

The predictability of which *Aux/IAA* gene member might carry the mutation conferring 2,4‐D resistance in any given species is obfuscated by the size of the gene family. With *IAA16* in *K. scoparia* and *Chenopodium album*, *IAA2* in *S. orientale* and now *IAA20* in *S. oleraceus* likely being the basis for 2,4‐D resistance contrasts to glyphosate resistance, for example, where the same EPSPS mutations are found across multiple species.^38^, ^39^ There appears to be flexibility in which Aux/IAA isoforms confer auxinic herbicide resistance depending on the species being studied. Implicit in this would be the interactions between different *Aux/IAA* gene members and auxin herbicides, and hence the possibility of different *Aux/IAA* mutations. Understanding this would be critical in the deployment of alternative auxin herbicides to control 2,4‐D‐resistant populations.

## 5 CONCLUSION

In this study, two deletions either side of the degron in *SoIAA20* were identified in eight populations of *S. oleraceus* with resistance to 2,4‐D, but not in three susceptible populations. These populations also had cross‐resistance to the other auxinic herbicides, including dicamba, chlopyralid and fluroxypyr, significantly reducing the herbicide options available for control of this weed in cropping systems. We suggest the deletions in SoIAA20 restrict 2,4‐D movement throughout the plant by trapping it in the SCF^TIR1/AFB^–2,4‐D–SoIAA20 complex, ultimately conferring 2,4‐D resistance. 2,4‐D is translocated out of the treated leaf of susceptible plants after treatment, but not for resistant plants. Molecular docking analyses of the SoIAA20 with its corresponding TIR1 in complex with IAA or 2,4‐D could shed light on how these mutations are affecting interactions that ultimately could lead to restricted 2,4‐D movement out of the cell. We will be studying *SoIAA20* transcript levels to better understand the response variability to 2,4‐D in the resistant populations as well. Better understanding the interactions between auxinic herbicides and these mutations could provide new information that could help identify herbicides that may still control these resistant populations.

## Supporting information


**Data S1.** Supporting Information.

## Data Availability

The data that support the findings of this study are available from the corresponding author upon reasonable request.
