# Supplementary material for: A novel mutation in SoIAA20 confers cross‐resistance to 2,4‐Dichlorophenoxyacetic acid and other auxinic herbicides in Sonchus oleraceus
Source: Pest Manag Sci. 2024 Sep 13;81(1):141–8. doi: 10.1002/ps.8413 (PMC11632205; doi:10.1002/ps.8413)
Supplement: Supplementary file 1 — Data S1. Supporting Information. [file PS-81-141-s001.docx]

Supplementary table 1: Sample locations with year, survival at 228 g/ha and presence of *SoIAA20* deletions

| SampleID | Survival at 228g/ha | SoIAA20 deletion | Location description | Region (year collected) |
| --- | --- | --- | --- | --- |
| Crocket | Y | Y | Liverpools district | Northern NSW (2014) |
| Yellow | N | N | Liverpools district | Northern NSW (2014) |
| White | N | N | Liverpools district | Northern NSW (2014) |
| S | N (dose response) | N (dose response) | Garden Island | SA (2015) |
| R1 | Y (dose response) | Y (dose response) | Samples sent in by farmer 1 | Southeast SA (2015) |
| R2 | Y (dose response) | Y (dose response) | Samples sent in by farmer 2 | Southeast SA (2015) |
| R3 | Y (dose response) | Y (dose response) | Samples sent in by farmer 3 | Southeast SA (2015) |
| 17-SE-213-C | Y | Y | The Gap | Southeast SA (2017) |
| 17-SE-215-C | Y | Y | Frances | Southeast SA (2017) |
| 17-SE-229-C | Y | Y | Bool Lagoon | Southeast SA (2017) |
| 17-SE-232-C | Y | Y | Lochabar | Southeast SA (2017) |

Supplementary table 2: % of 2,4-D that has moved to the rest of the shoot (RoS) from treated leaf at 72 HAT in S and three R populations with standard error (SEM) on top section. Below section has Tukey’s comparison test from a one-way ANOVA for S and three R populations of 2,4-D that has moved to the rest of the shoot from treated leaf at 72 HAT.

| Population (n= no. of plants) | % of 2,4-D translocated from treated leaf |  |
| --- | --- | --- |
|  |  |  |
| S (n=11) | 5.22 ± 0.99 a |  |
| R1 (n=12) | 1.57 ± 0.41 b |  |
| R2 (n=12) | 2.98 ± 0.49 b |  |
| R3 (n=12) | 2.23 ± 0.24 b |  |
| Tukey's multiple comparisons test | Adjusted P-Value |  |
| S vs. R1 | S (0.0004) |  |
| S vs. R2 | S (0.0482) |  |
| S vs. R3 | S (0.0045) |  |
| R1 vs. R2 | NS (0.3179) |  |
| R1 vs. R3 | NS (0.8479) |  |
| R2 vs. R3 | NS (0.7927) |  |

Supplementary table 3: Primer sequences for PCR amplification of *Aux*/*IAA* gene candidates in *S.oleraceus* with their closest *L.sativa* match (GID) based on highest bit-score in NCBI BLAST search.

| Aux/IAA gene candidate names | Forward primer 5'-3' | Reverse primer 5'-3' | Close Aux/IAA match GID |
| --- | --- | --- | --- |
| AUXIAA mRNA 1 | ATGGAAAGTGGGTATTCGAG | GTACATATGTTGGAAATCTCAG | 111907942 |
| AUXIAA mRNA 2 | ATGAATATGGAAGACGGATTG | GCTTCTGATCCTTTCATTATTC | 111883505 |
| AUXIAA mRNA 3 | ATGGAGGGATACCCTAAATTG | CAGATGTCTTCAACACACGAAG | 111906099 |
| AUXIAA mRNA 4 | GGATTGCAGTCTTGACTTAC | TCCAACACCCATGACTTC | 111909856 |
| AUXIAA mRNA 5 | ATGGAGCTTCAACTGGC | GGCGTTGAACTGTCTCG | 111877814 |
| AUXIAA mRNA 6 | GTCTGAGCCACTAGAACAC | CTAGTCCAGTTGCTTCGG | 111909089 |
| AUXIAA mRNA 7 | GGCGAAAATAACCAACAACC | CCTAAACCGGTTGCATCTG | 111883851 |
| AUXIAA mRNA 8 | GTCTGAGCCACTAGAACACG | GGCTAGTCCAGTTGCTTC | 111909089 |
| AUXIAA mRNA 9 | GACAGAACATGCCGAAAT | GTTTAAGGCGTTGAACTG | 111877814 |
| AUXIAA mRNA 10 | GACGTCGTTTGAGGAGAC | CTTACCAAGCCCAATAGC | 111881233 |
| AUXIAA mRNA 11 | GGAACTTGAGCTTAGTCTTTC | GGTAGAAAAGATGCATACTCC | 111898411 |
| AUXIAA mRNA 12 | GGACGTTACCCTTGGCTTG | CCGTACTGCCAAGGAAC | 111881242 |
| AUXIAA mRNA 13 | GTCATCGGAGACGTCC | GCTAGTCCATTAATGGCTTC | 111910248 |
| AUXIAA mRNA 14 | GATGCACCTCAAGGAAAC | CTAGTCCAATTGCATCGGA | 111877048 |
| AUXIAA mRNA 15/Putative *SoIAA20* | GGAGCTTCATTTGGGTCT | GTTTCTTCACCAATTTCAGAC | *LsIAA20*/111878246 |
| AUXIAA mRNA 16 | GGATCAAAAGAGAACTTCTTTG | TCTTGCTTCTGAACCCTTC | 111915100 |
| AUXIAA mRNA 17 | GAGCTTCATTTGGGTCTTG | CTTCAACAATTTCAGACGTTT | 111878246 |
| AUXIAA mRNA 18 | GACCTCAAGGACTAGGGC | CTACGTATCTGAGCTCTTCA | 112509386 |
| AUXIAA mRNA 19 | GTCCATTGAAGAACATGATTAC | CATATCATCACTAGACCAATTGC | 111907077 |
| AUXIAA mRNA 20 | GATGCACCTCAAGGAGAC | CAATCGCATCCGATCCTT | 111885465 |
| AUXIAA mRNA 21 | GACTCCTCCGCTGTTAG | GTTGACTAAACCAATTGCATC | 111891391 |
| AUXIAA mRNA 22 | CAACTTTCCGACTAGGAAGA | CAAGATTGGTATGGAATTGCT | 111885963 |
| AUXIAA mRNA 23 | GATCTCAACTACAAAGCAACC | CTCTTGCTTCATACCCTTTCA | 111910911 |
| AUXIAA mRNA 25 | GGATCAAAAGAGAACCTCTTTG | GAATCACCCAACCCTCGT | 111915100 |
| AUXIAA mRNA 26 | CATTGGAGACGTCCAAAATC | CAGCTAGAGATGAAGCATTTTG | 111910248 |
| AUXIAA mRNA 27 | GGATTGCAGTCTTGACTTAC | CGACACACCAACGCATC | 111909856 |
| AUXIAA mRNA 28 | GTCTGTGCCACTCGAAC | CTACCTAGCCCAATTGCTTC | 111910288 |
| AUXIAA mRNA 29 | GAACATGGAAGACGGATTG | GTTGCATCCCAATCCTTTTG | 111883505 |
| AUXIAA mRNA 30 | GTCTGTGCCACGGGA | CGTTCCTAATCCGATTGC | 111900813 |
| AUXIAA mRNA 31 | GGATCTAATTAATTTCGAAGCTAC | CTCTCCATAACTTCAGAACCTC | 111881263 |
| AUXIAA mRNA 32 | GACGTCGTTCGAGGAG | CTTACCAAGCCCAATAGCT | 111881233 |
| AUXIAA mRNA 33 | GAAAGTGGGTATTCGAGAG | CACTCGCAGCCTCTTAAC | 111907942 |
| AUXIAA mRNA 34 | GCACCTAAAGGAGACTGAG | CCCAATCGCATCCGATC | 111885465 |
| AUXIAA mRNA 35 | GATGCACCTCAAGGAAAC | CTAGTCCAATTGCATCTGATC | 111877048 |
| AUXIAA mRNA 36 | GTCTTCACCGCTGTTAGC | CTAATCCGATTGCATCTGAG | 112515126 |
| AUXIAA mRNA 37 | CGAAGCAACCGAGCTAC | CTCTCCATAACTTCAGAACCTC | 111881263 |
| AUXIAA mRNA 38 | GTCCATTGAAGAACATGATTAC | CTAGACCAATTGCATCTGAAC | 111907077 |
| AUXIAA mRNA 39 | CTGTGCCACTCGAACATG | CTAGCCCGATTGCTTCTG | 111910288 |
| AUXIAA mRNA 40 | GTCTTCACCTCTGTTAGCC | CCAATTGCATCTGAACCC | 112515126 |
| AUXIAA mRNA 41 | GATTATGAAAGGTGTTGAAGG | GTTGACTAAACCAATTGCATC | 112515126 |
| AUXIAA mRNA 42 | GGGACCTCAAGGACTAGG | CGTGTAAAAAGATTTACCAATG | 111918948 |
| AUXIAA mRNA 45 | TCTGTGCCACGGGAAC | GATTGCTTCGGAGCCTTTC | 111900813 |
| AUXIAA mRNA 46 | GGATCTCAATTACAAAGCAAC | CAAATCCAACCCTCTTGCT | 111910911 |

Supplementary table 4: LD50 values for all populations with and without malathion with confidence intervals in parentheses; F-test with P-values in parentheses for the curves of the populations with and without malathion. Where a low P-value score denotes a significant difference between the malathion and non-malathion treatment.

| Treatments | LD50 with confidence intervals | Sum-of-squares F-test (P-value) |
| --- | --- | --- |
| S with malathion | 59.75 (48, 74) | S (0.04) |
| S without malathion | 57.01 (35, 85) |  |
| R1 with malathion | 789.06 (508, 1292) | NS (0.12) |
| R1 without malathion | 1605 (983, 3737) |  |
| R2 with malathion | 394.8 (195, 743) | NS (0.83) |
| R2 without malathion | 325.9 (135, 667) |  |
| R3 with malathion | 183.6 (123.5, 268.6) | NS (0.75) |
| R3 without malathion | 200.1 (136, 291.6) |  |


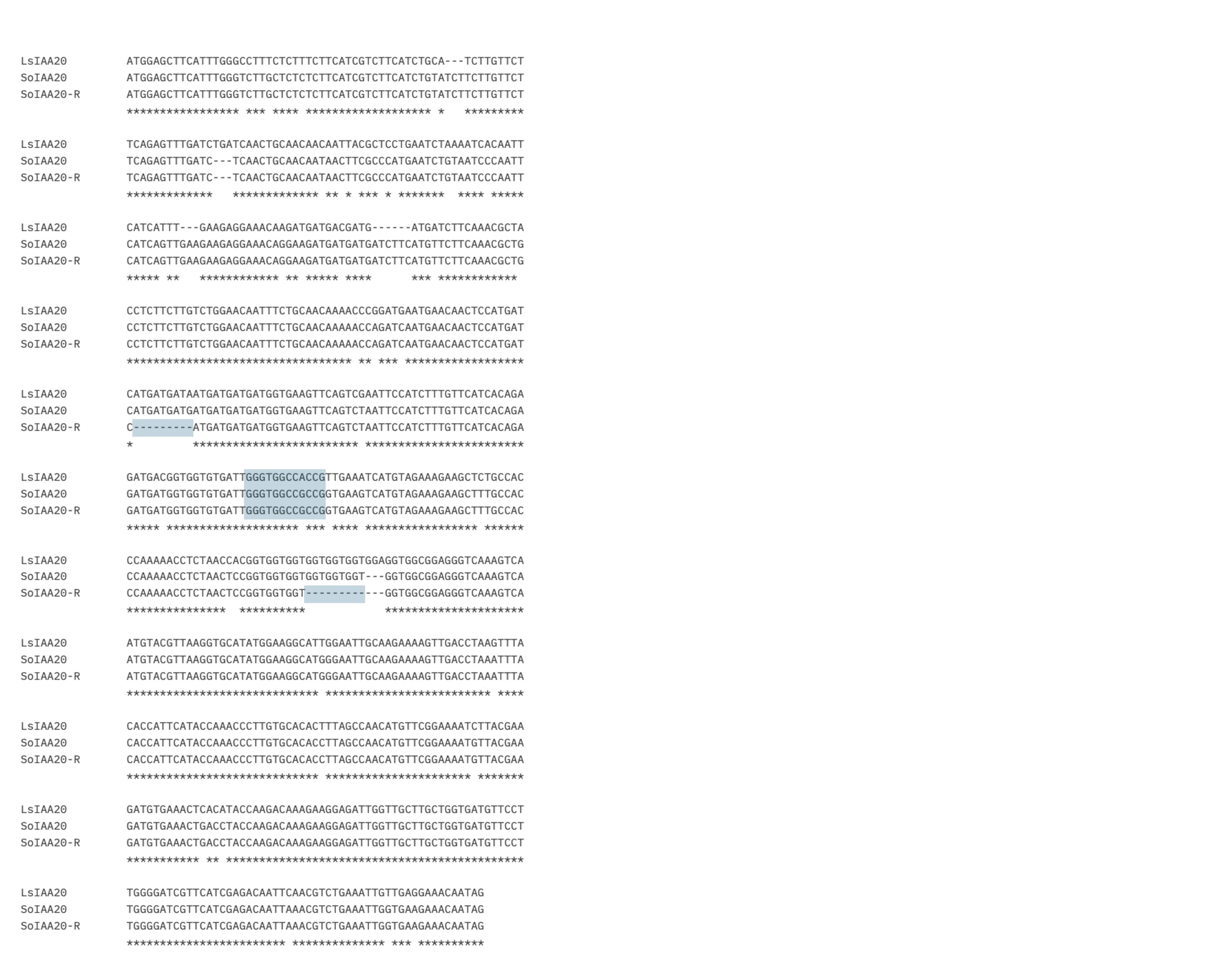


Supplementary figure 1: A MUSCLE nucleotide alignment of the full cDNA of *SoIAA20* and the resistant allele, *SoIAA20-R*, with its *L. sativa* counterpart, *LsIAA20*. Grey box highlights the deletions and the GWPP degron core.
